# Elevated levels of arginase activity are related to inflammation in patients with COPD exacerbation

**DOI:** 10.1186/s12890-021-01629-w

**Published:** 2021-08-21

**Authors:** Marcel Jose Rodríguez-Guzmán, Germán Peces-Barba Romero, Sandra Pérez Rial, Cristina Serrano del Castillo, Miguel Ángel Palomero Rodríguez, Ignacio Mahillo-Fernandez, Felipe Villar-Álvarez

**Affiliations:** 1 Pulmonology Department, IIS Jiménez Díaz Foundation, CIBERES, Ave Reyes Catolicos #2, 28040 Madrid, Spain; 2 Immunology Department, Central Laboratory-Flow Cytometry Section, Jiménez Díaz Foundation, Madrid, Spain; 3 Anesthesiology and Resuscitation Department, Ramón y Cajal Hospital, Madrid, Spain; 4 Statistics Department, IS Jiménez Díaz Foundation, CIBERES, Madrid, Spain

**Keywords:** COPD, COPD exacerbation, Arginase activity, Inflammation, Remodelling

## Abstract

**Introduction:**

Within the pathogenesis of the chronic obstructive pulmonary disease (COPD) there are interactions between different inflammatory mediators that are enhanced during an exacerbation. Arginase is present in bronchial epithelial cells, endothelial, fibroblasts and alveolar macrophages, which make it a probable key enzyme in the regulation of inflammation and remodelling. We aimed to find a potential relationship between arginase activity, inflammatory mediators in COPD patients in stable phase and during exacerbations.

**Methods:**

We performed a prospective, observational study of cases and controls, with 4 study groups (healthy controls, stable COPD, COPD during an exacerbation and COPD 3 months after exacerbation). We measured arginase, inflammation markers (IL-6, IL-8, TNF-∝, IFN-γ and C reactive protein), and mediators of immunity: neutrophils, monocytes, total TCD3 + lymphocytes (CD3ζ), CD4 + T cells, CD8 + T cells, NK cells.

**Results:**

A total of 49 subjects were recruited, average age of 69.73 years (59.18% male). Arginase activity is elevated during an exacerbation of COPD, and this rise is related to an increase in IL-6 production. The levels of IL-6 and IL-8 remained elevated in patients with COPD at 3 months after hospital exacerbation.

We did not find a clear relationship between arginase activity, immunity or with the degree of obstruction in COPD patients.

**Conclusions:**

Arginase activity is elevated during an exacerbation of COPD, and it could be related to an increase in the production of IL-6. Levels of IL-6, IL-8, and arginase activity remain elevated in patients with COPD at 3 months after hospital exacerbation. Arginase activity could contribute to the development of COPD.

## Introduction

Inflammation is one of the first responses of the body's immune system to any aggression, such as that caused by tobacco smoke and other inhaled contaminants [1]. Under normal conditions, the activation of acquired immunity mediated by lymphocytes establishes a barrier against the expansion of a lesion, and the recovery of damaged tissue is promoted. However, in chronic obstructive pulmonary disease (COPD) this balance is impaired, which leads to changes in the structure of the airways, due to destruction of the alveolar spaces and the pulmonary vasculature [2].

Patients with stable COPD have elevated serum levels of circulating pro-inflammatory mediators, such as C-reactive protein (CRP), fibrinogen, interleukin (IL)-6, and IL-8 [3], and there is an increase in T lymphocytes in the lung, predominantly CD8+ (cytotoxic) [4]. The numbers of CD4+ lymphocytes are also increased, which are differentiated to helper T cells type 1 (Th1), with the stimulus of IL-12. Th1 cells are responsible for the production of interferon-gamma (IFN-γ), a probable cause of emphysema [5].

During an exacerbation, the mechanisms of pulmonary and systemic inflammation are directly involved. Host defence factors against different pathogens may also play a role in individual predisposition to exacerbation [6]. Systemic inflammation increases in exacerbation and, although the causes of this response in COPD are unclear, it seems to be an overflow of the markers of inflammation (CRP, IL-8, IL-6) from the lungs [7, 8].

After an exacerbation, some patients take time to return to baseline functional grade levels of the stable phase. Up to 23% of patients did not recover from worsening symptoms until 35 days after, and had persistently high levels of CRP. The levels of IL-6 and IL-8 and tumor necrosis factor-∝ (TNF-∝) are elevated as part of the systemic inflammation in exacerbations [9].

Arginase is an enzyme that competes with the isozymes of nitric oxide synthase (NOS), for amino acid L-Arginine, catalyzing its hydrolysis to L-ornithine and urea [10]. Both isoforms, (I and II), are expressed in bronchial, endothelial, fibroblast and alveolar macrophage epithelial cells. Arginase II is also expressed in lung parenchyma cells [11]. The macrophages are induced to produce arginase I due to the action of several cytokines related to the helper T cells type 2 (Th2). As arginase I is active, the action of NOS is inhibited, mainly NOS subtype 2 or inducible NOS (iNOS).INF-ỵ, produced mainly by Th1 cells, induces the action of iNOS, thus increasing nitric oxide production [12].

During the early stages of the inflammatory response the activation of polymorphonuclear has a pro-inflammatory effect, which is accompanied by an anti-inflammatory process that would contribute to the resolution through the release of arginase, decreasing the levels of arginine and inhibiting the functions of T cells. This inhibition is caused by a decrease in the expression of the CD3zeta (CD3ζ) chain of the T lymphocyte receptor [13].

The increase in the expression of iNOS in COPD is implicated in the regulation of airway tone and functional airflow limitation, while the activity of arginase I is related to an increase in the sensitivity of airways [14]. Human neutrophils express high levels of arginase I in azurophilic granules, which could be released in patients with COPD [15].

An increase in arginase activity results in an increase in the production of L-ornithine and all its products (L-proline, L-glutamate, spermidine), which can play an important role in the remodeling and development of fibrosis in diseases like COPD [15]. However, the role that arginase plays in COPD has not been established yet. We hypothesized that arginase activity is related to inflammation and the alteration of adaptive immunity in patients with COPD in a stable and acute phase. Our main objective was to identify new pathogenic mechanisms, triggered by arginase activation, in patients with COPD in the stable and acute phases. We aimed to find a potential relationship between arginase, mediators of immunity and inflammation such as CRP, IL-6, IL-8, TNF-∝ and IFN-γ in COPD patients in stable phase and during exacerbations.

## Methodology

Prospective, observational, case-control study. All subjects included were from both sex, over 40 years old. Four study groups: Healthy subjects, without COPD, relatives of patients and hospital workers who met de inclusion and exclusion criteria (control, group I). Patients with COPD with 10 or more pack-year history in a stable phase (stability of symptoms and without exacerbations in the last three months), with less than two exacerbations in the last year (stable COPD, group II). COPD patients, with a smoking history of 10 pack-years or more admitted in hospital due to an exacerbation (exacerbated phase COPD, group III). In this group, measurements were done during the exacerbation. After the exacerbation, the measurements were repeated when the patient reached the stability phase, which was considered 3 months after the exacerbation. This fourth group consisted of COPD patients who underwent measurements three months after the exacerbation (COPD patients, 3 months after an exacerbation, group IV). The COPD patients were diagnosed according to GOLD criteria [16].

Exclusion criteria for the study groups were the presence of other chronic pulmonary obstructive disorders, chronic inflammatory diseases, previous surgeries one month prior the recruitment, or HIV positive patients. Moreover, chronic administration of systemic corticosteroids at unstable daily doses (less than six weeks with stable doses) or a daily dose of more than 10 mg of prednisone or its equivalent, the presence of cancer of any organ, radiotherapy or chemotherapy in the last 5 years and treatment with an immunosuppressant or cytostatic drug.

### Variables and measurements

Age, weight, height, and smoking history data were collected. Modified dyspnea scale of the Medical Research Council (mMRC) [17] and the COPD Assessment Test (CAT) [18] were applied. Spirometry with a post-bronchodilator test was used, in accordance with the ATS/ERS standard of 2005 [19].

For the measurement of peripheral blood immunophenotype and immunophenotypic studies of lymphocyte populations were performed (TCD3+ lymphocytes, TCD4+ subpopulation, TCD8+ subpopulation, NK lymphocytes), with the determination of CD3ζ chain expression in T lymphocytes and study of monocyte. T lymphocytes were identified based on size and granulation along with the expression of the CD3 marker, and their distribution in TCD4+ and TCD8+ cells. The percentage of TCD3 cells expressing CD3ζ has also been calculated. NK cells were recognized based on size and granulation together with the expression of CD16 and/or CD56 in the absence of the CD3 marker and the percentage of these cells expressing TCRζ was also analyzed. Monocytes were identified on the basis of their size and granulation differentiating classic and non-classic monocytes based on the expression of CD16.

For the measurement of peripheral blood lymphocyte and monocytes subjects, a multiparametric flow cytometry study was performed. Identification of total T lymphocytes was based on their forward and side scatter characteristics and CD45 signal. Lymphocyte subpopulations were analyzed according to their forward and side scatter characteristics and the following immunophenotype: Total T Lymphocytes: CD3+CD45++; T Helper Lymphocytes: CD3+CD4+CD45++; T Cytotoxic Lymphocytes: CD3+CD8+CD45++ and NK cells: CD56+CD16+CD3-CD45++. The percentage of TCD3 cells expressing CD3ζ was also determined in T and NK populations. Identification of total neutrophils, eosinophils and monocytes were based on their forward and side scatter characteristics and CD45 signal. Classic and non-classic monocytes distributions were identified based of the expression of CD16 and CD14 in this population.

The measurement of arginase activity was performed according to the protocol previously described in the literature [20]. The sonication protocol for cell lysis was followed. We measured the hydrolysis of arginine by converting L-Arginine to urea. One unit of enzymatic activity was defined as the amount of enzyme needed to catalyse the formation of 1 μmol urea/min.

The statistical analysis was performed with the statistical program STATA Data analysis and statistical software. Version 11. StataCorp LLC. Qualitative variables were described with the use of absolute and relative frequencies. The variables studied were divided into quantitative with normal distribution, quantitative with non-normal distribution, and qualitative. Groups were compared two by two in relation to each of the analysis variables.

Quantitative variables with normal distribution were described with mean and standard deviation. The normality of these variables was contrasted with the Kolmogorov-Smirnov test. Comparisons were made with the use of the Student's t test.

Quantitative variables with non-normal distribution were described with the help of the median and interquartile range and were compared using the Mann-Whitney test. To evaluate the relationships among quantitative variables, the Spearman correlation coefficient was calculated.

Qualitative variables were compared using the Chi-square test, or Fisher's exact test when the approximation to the Chi-square distribution was not correct.

To study which variables could be independently related to a given response, multivariate linear regression models were used. These models were summarized with the estimation of the coefficients, together with their 95% confidence intervals, their p-values and with the adjusted R squared.

## Results

**Fig. 1:**
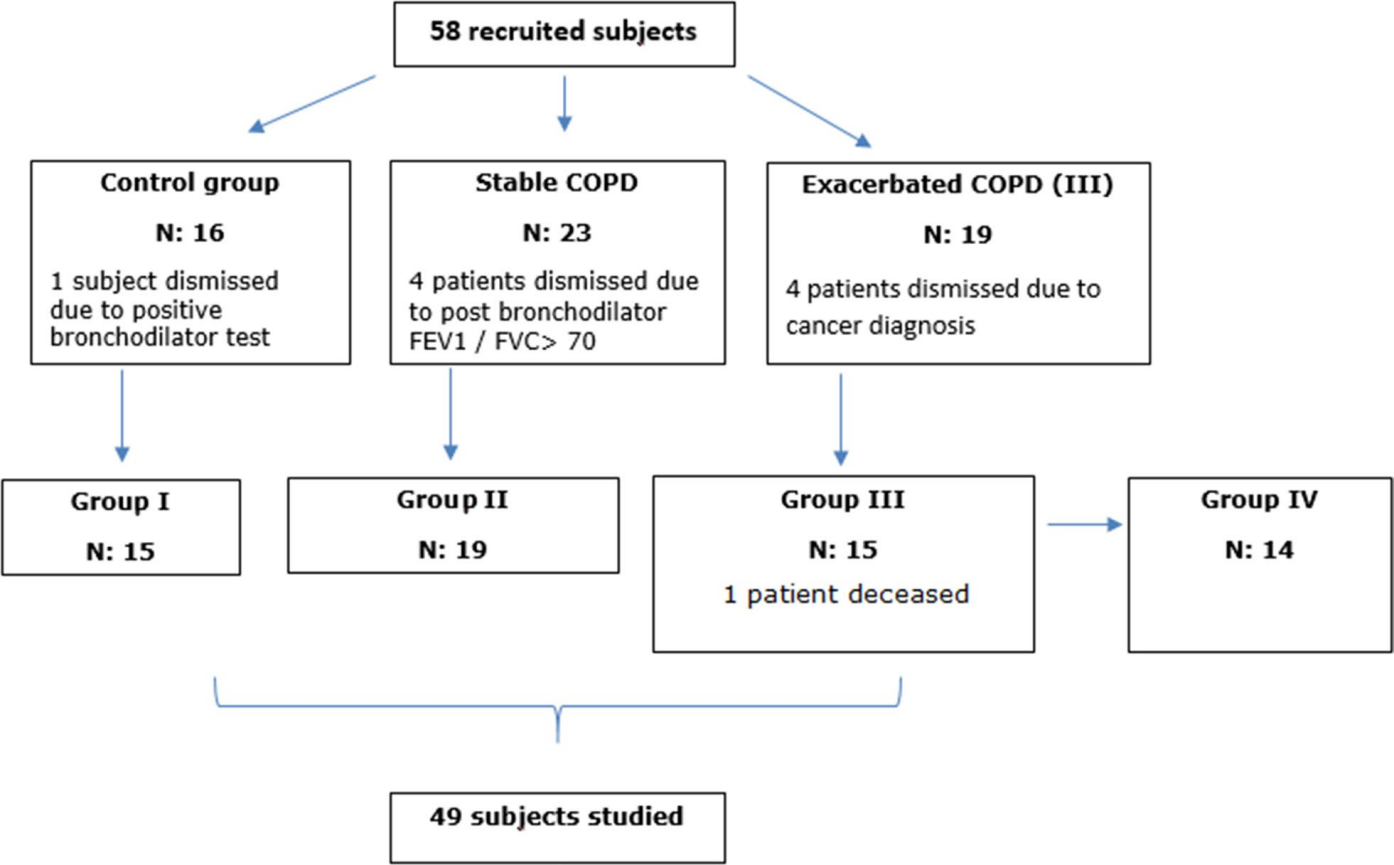
Population distribution in the different study groups. *Group I: Control; Group II: stable COPD; Group III: exacerbated phase COPD. The subjects in the fourth group: COPD 3 months after exacerbation (IV), are the same than the ones from group III

58 subjects were recruited. After reviewing the inclusion and exclusion criteria, 49 subjects were selected as the study population for the analysis of the results. Population distribution in the 4 study groups is detailed in Fig. 1. The average age of the population was 69.73 years (59.18% men). Similar values were observed in all the groups, without significant differences in age, anthropometric characteristics, and smoking (Table 1). Most subjects had a smoking history (84%). Group III had a lower BMI and a higher score on the mMRC scale than the stable phase COPD (*p* 0.032 and 0.002, respectively). The exacerbation cause in group 3 was due to infection in 10/15 subjects (66.67%). In 5/15, an infectious cause was not determined nor suspected. 2 of them were active smokers, a known trigger for COPD exacerbations. One of the patients died due to the exacerbation.

**Table 1:** Characteristics of the population studied

	Group (I)^a^	Group (II)^a^	Group (III)^a^	Group (IV)^a^	*p* I + II	*p* I + III	*p* II + III	*p* III + IV	*p* I + IV
Age (years)	67.67 (10.34)	69.37 (8.65)	71.40 (11.34)	70.64 (11.37)	0.605	0.467	0.717	0.859	0.557
BMI (kg/m^2^)	28.67 (5.94)	28.14 (3.74)	25.45 (3.100)	25.36 (3.375)	0.752	0.076	0.036	0.943	0.032
Pack-year	39.14 (20.25)	59.06 (25.91)	51.73 (20.00)	52.21 (20.67)	0.082	0.185	0.426	0.950	0.378
CAT	7.47 (3.58)	13.53 (4.41)	15.73 (3.45)	17.14 (3.439)	< 0.001	< 0.001	0.016	0.281	0.122
mMRC < 2^b^	15 (23.8%)	5 (7.9%)	1 (1.60%)	2 (3.2%)	< 0.001^b^	< 0.001^b^	0.002^b^	0.770^b^	0.002^b^
mMRC ≥ 2^b^	0 (0%)	14 (22.2%)	14 (22.2%)	12 (19.00%)					
FEV_1_pb (%)	121.40 (19.88)	58.15 (21.87)	47.51 (13.87)	76.09 (21.37)	< 0.001	< 0.001	0.157	0.932	0.111
FEV_1_/FVCpb	81.05 (6.322)	48.06 (12.10)	45.64 (11.14)	47.53 (9.199)	< 0.001	< 0.001	0.892	0.624	0.553
Dlco (%)	93.96 (24.71)	81.21 (22.55)	68.49 (14.26)	59.39 (11.57)	0.133	< 0.001	0.001	0.125	0.155
FeNO (ppb)	25.86 (16.41)	17.93 (23.31)	14.30 (8.455)	13.20 (6.704)	0.289	0.016	0.424	0.809	0.796

Data expressed as mean and standard deviation (quoted) unless otherwise indicated

BMI, body mass index; Pack-year, number of packs of cigarettes smoked per day by the number of years the person has smoked; CAT, COPD assessment test; FVCpb, forced post-bronchodilator vital capacity; FEV_1_pb, Forced expiratory volume in the first second post-bronchodilator; FEV_1_/FVCpb, FEV_1_/FVC post-bronchodilator; Dlco, diffusion capacity of carbon monoxide; FeNO, nitric oxide in exhaled air; mMRC, modified dyspnea scale of the Medical Research Council

^a^Group I: Control; Group II: stable COPD; Group III: exacerbated phase COPD. The subjects in the fourth group: COPD 3 months after exacerbation (IV), are the same than the ones from group III

^b^Qualitative variable. The values of frequencies and percentages in relation to the entire population are shown

We found an increase of the total leukocytes, the percentage of neutrophils, and the value of the CRP in group III; with a significant difference compared to other groups (*p* <0.05).

No differences were discovered in arginase activity between groups I and II. IL-6 levels were decreased in patients with stable COPD (*p* 0.001). The IFN-γ value was also high in the stable phase COPD group, without reaching statistical significance (*p* 0.051). Almost all immunophenotype values were similar in both groups, except for CD8+ lymphocyte levels, which were significantly increased in stable COPD (Table 2).

**Table 2:** Comparison of arginase activity, mediators of inflammation and peripheral blood immunophenotype, between the different groups

	N	Group (I)^a^	Group (II)^a^	Group (III)^a^	Group (IV)^a^	*p* I + II	*p* I + III	*p* II + III	*p* III + IV	*p* II + IV
Actarginase (µmol urea/min)	49	0.500 (0.122)	0.521 (0.217)	0.70 (0.33)	0.59 (0.32)	0.720	0.042	0.067	0.357	0.483
CRP (mg/L)	49	0.551 (0.685)	0.689 (0.750)	1.283 (1.200)	2.602 (3.110)	0.445	0.027	0.061	0.038	0.147
IL-6 (pg/ml)	37	13.45 (1.992)	10.74 (1.040)	14.54 (4.87)	15.57 (2.58)	0.001	0.522	0.037	0.643	0.005
IL-8 (pg/ml)	37	64.53 (17.13)	53.49 (9.65)	69.86 (15.46)	70.89 (17.70)	0.081	0.475	0.008	0.904	0.018
TNF-a (pg/ml)	37	100.0 (12.72)	98.54 (12.95)	95.70 (10.56)	94.72 (18.75)	0.797	0.421	0.591	0.902	0.660
INF-γ (pg/ml)	37	58.22 (8.285)	75.62 (25.29)	89.30 (9.97)	87.42 (4.96)	0.051	< 0.001	0.121	0.675	0.162
Neutrophils (%)	49	61.57 (8.13)	62.12 (7.60)	78.76 (12.93)	62.78 (10.18)	0.841	< 0.001	< 0.001	0.001	0.832
Lymphocytes (%)	49	28.85 (7.49)	27.77 (7.35)	15.31 (9.48)	27.47 (8.92)	0.675	< 0.001	< 0.001	0.001	0.917
CD3 (%)	49	72.25 (7.01)	72.02 (8.90)	66.46 (12.67)	73.07 (9.64)	0.935	0.135	0.142	0.127	0.749
CD4 (%)	49	48.66 (8.64)	42.90 (11.72)	41.01 (9.83)	42.46 (8.21)	0.122	0.032	0.619	0.670	0.905
CD8 (%)	49	22.21 (9.32)	29.02 (9.99)	25.00 (6.75)	29.82 (11.60)	0.050	0.355	0.192	0.179	0.832
MC CD16+ (%)	49	21.20 (7.57)	23.79 (13.63)	17.00 (9.07)	18.00 (9.84)	0.488	0.186	0.116	0.786	0.199
MC CD16− (%)	49	78.87 (7.41)	78.00 (12.01)	83.93 (9.97)	83.36 (10.64)	0.809	0.131	0.147	0.885	0.199
CD3ζ (%)^b^	49	77.58 (22.42)	69.39 (40.75)	64.86 (46.75)	68.36 (43.75)	0.382	0.291	0.699	0.773	0.913
NKCD56++ (%)^b^	49	4.24 (3.74)	4.63 (4.76)	2.13 (2.31)	3.18 (3.40)	0.812	0.089	0.047	0.237	0.338
CD3ζNK (%)^b^	49	94.20 (1.00)	98.61 (0.00)	81.12 (29.55)	93.48 (14.75)	0.330	0.174	0.053	0.169	0.061
Monocyte (%)^b^	49	6.44 (2.38)	6.626 (2.70)	4.580 (3.55)	8.76 (1.35)	0.759	0.026	0.013	0.127	0.415

Data expressed as mean and standard deviation unless otherwise indicated

IL-6, Interleukin 6; IL-8, Interleukin 8; IFN-γ, Interferon gamma; TNF-∝, Tumor necrosis factor alpha; ActArginase, arginase activity, MC CD16+, CD16+ monocytes (non-classical); MC CD16−, Monocytes CD16− (classic)

^a^Group I: control; Group II: stable COPD; Group III: exacerbated phase COPD. The subjects in the fourth group: COPD 3 months after exacerbation (IV), are the same than the ones from group III

^b^Variables with non-normal distribution: the median value and the inter-quartile range are shown in parentheses. Comparison with the Mann–Whitney test

Patients with COPD in group II, when compared with group I, presented high neutrophil values, as well as a decrease in the percentage of total lymphocytes, in the sub-group of CD4+ lymphocytes and in the percentage of monocytes. Arginase activity was observed to be significantly elevated (*p* 0.042) in this group. When comparing both groups we also noted an increase in IFN-γ levels (*p* <0.001) (Table 2).

**Fig. 2:**
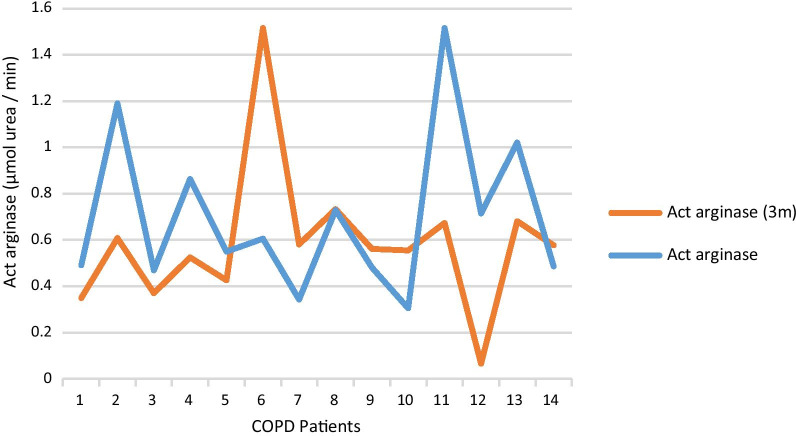
Change in arginase activity in exacerbated group and 3 months after exacerbation. Act arginase: arginase activity in exacerbated phase (group III); Act arginase (3 m): arginase activity exacerbated group 3 months after exacerbation (group IV). Actarginase measured in µmol urea/min

Arginase activity was increased during an exacerbation compared to stable phase, but did not reach significance (*p* 0.067). It is worth noting that when comparing arginase activity during an exacerbation and three months after it, we didn’t find significant changes (Figure 2). IL-6 and IL-8 are significantly increased during an exacerbation (*p* 0.037 and 0.008 respectively). We did not observe differences in the value of TNF-∝ between the two groups. The percentage of lymphocytes, monocytes, and NK cells was decreased in group III. The percentage of the CD3ζ chain expressed by NK cells was also lower in group III, but this decrease was not statistically significant (Table 2).

When comparing groups III and IV, we noticed a higher percentage of neutrophils and a decrease in the percentage of lymphocytes during the exacerbation. Arginase level remained elevated in group IV, although not significantly. The rest of the percentage values of the studied cells, no significant differences were detected (Table 2).

There was an increase in serum levels of IL-6 and IL-8 in COPD subjects in group IV when compared with group II. There were no differences in the peripheral blood immunophenotype markers. Arginase activity had no significant changes between these two groups (Table 2).

**Fig. 3:**
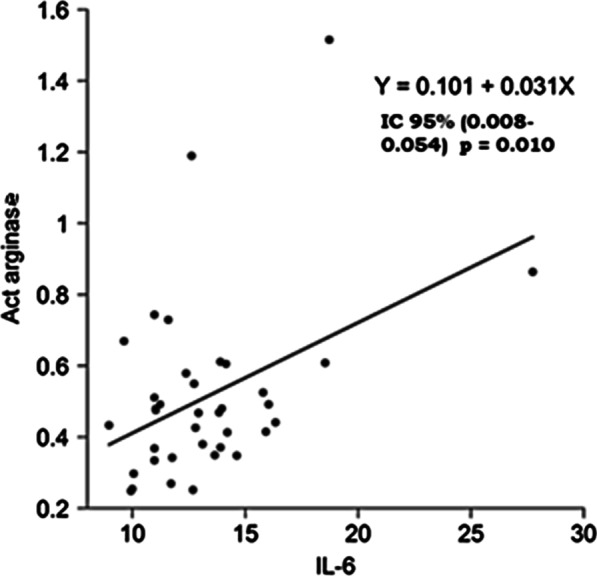
Relationship between arginase activity and IL-6 after multivariable study. Act arginase: arginase activity (µmol urea/min); IL-6: Interleukin 6 (pg/ml)

In the multivariable study, a direct relationship was observed between arginase activity and IL-6 levels, (95% confidence interval of 0.008–0.054, *p* 0.010) (Figure 3). No correlation was noticed among arginase activity, the percentage of eosinophils, and the different lymphocyte cell markers in peripheral blood (Table 2). We didn’t find a relationship between arginase activity and CRP levels, nor a relationship between the value of arginase activity and forced expiratory volume in 1 second (FEV1) in patients with stable COPD (*p* 0.617).

An inversely proportional relationship between IL-6 levels and the percentage of neutrophils (slope of −0.442, 95% confidence interval: −0.731, −0.152, *p* 0.003) was noted.

## Discussion

Arginase activity is elevated during an exacerbation of COPD, and it could be associated with an increase in the production of IL-6. Comparing COPD patients in stable phase with the COPD group during an exacerbation, we observed that the latter had an increase in arginase activity, although it did not reach statistical significance. This increase during an exacerbation can be explained by the augmented neutrophilic activity in the initial phase of infection, in the case of exacerbations of infectious cause. Darcy et al detected circulating neutrophils expressing arginase in septic patients, the number of which was proportional to the severity of the disease [21]. They also found an increase in arginase activity, which related to a decrease in T cell activation, secondary to a decrease in the availability of L-Arginine [21].

We found a direct relationship between the value of IL-6 and arginase activity. Gutierrez et al. [22], studied groups of COPD patients with exacerbation and exacerbation secondary to community-acquired pneumonia and observed a decreased value in the expression of IL-6 in the control group. They saw that the macrophages of patients with an exacerbation of COPD without pneumonia had an increase in the amount of arginase mRNA and in the induction of IL-6.

It has been described that alterations derived from the methylation of L-Arginine in patients with COPD produce a predisposition to metabolism via the arginase route, increasing its activity during an exacerbation. In this sense, asymmetric dimethyl arginine (ADMA) and symmetric dimethyl arginine (SDMA), metabolites that are significantly increased during COPD exacerbation, could be an early marker of an exacerbation [23]. This is why we believe that the determination of serum arginase activity could also be useful in the detection of an exacerbation in its initial stages. On the other hand, a study found a correlation between L arginine and its methylated residues with thrombo-inflammation in ischemic stroke. Also, a strong negative correlation was found between IL-6 and L-Arginine levels in the hyper acute phase in patients with post-stroke infection [24].

With regard to the relationship between arginase activity and immunity, we observed an expected increase in the percentage of neutrophils during an exacerbation, which was accompanied by a decrease in lymphocyte and monocyte levels. We found a decrease in the percentage of the CD3ζ chain expressed by NK cells in COPD patients during an exacerbation, compared with COPD in stable phase, as well as in COPD patients in group IV. However, both comparisons were not significant. It has been described that an increase in neutrophils in septic patients increases arginase expression and promotes suppression of T lymphocyte proliferation, seeing an inverse association between the number of neutrophils and the expression of the CD3ζ chain [21].

Another study compared serum levels of arginase I (measured by ELISA), myeloid suppressor cells (MDSCs) and levels of TCRζ and CD3 epsilon in patients with stable-stage COPD, ex-smokers and active smokers without obstruction measured by spirometry. In COPD patients and active smokers, the concentration of arginase I was similar and the values were significantly higher in relation to ex-smokers [25]. They also observed that smoking increased the activity of MDSCs, and their activity was associated with a significant decrease in the expression of TCRζ.

IL-6 is produced by several types of cells (macrophages, dendritic cells or B lymphocytes), and has a function linked to the control of the differentiation of CD4+ lymphocytes into Th2/Th1. When secreted, it potentiates differentiation to effector Th2 cells and inhibits differentiation to Th1 [26]. It is likely that this function of IL-6 contributes in regulating, in early stages of inflammation, the Th1/Th2 differentiation during the activation of CD4+ T cells [26]. On the other hand, IL-6 increases hepatic CRP production [27], which supports this theory of modulation of the onset of the acute phase.

In our study we have linked the presence of an increase in IL-6 with an increase in the activity of arginase in patients with COPD during an exacerbation. Macrophages are induced to produce arginase I by the action of several cytokines related to Th2 lymphocytes, such as IL-4, IL-10 and IL-13. As arginase I is active, the action of NOS is inhibited, due to depletion of L arginine [12]. Therefore, during an exacerbation, COPD patients have an increase in the production of IL-6. The differentiation of T lymphocytes to Th2 types is favored, augmenting the production of arginase and in turn the production of polyamines and collagen. Finally, the production of NO is decreased, leading to bronchial hyper reactivity.

It is possible that the inhibition of this enzyme could represent a future therapeutic target. In this sense, Pera et al [28] demonstrated the efficacy of an arginase inhibitor: 2 (S)-amino-6-boronohexanoic acid (ABH). In this study live guinea pigs were exposed to a bronchial-challenge with intranasal lipopolysaccharide (LPS). Those who were treated with ABH expressed a decrease in arginase activity in vivo, a decreased number of neutrophils, and a 75% decrease in the amount of hydroxyproline, an estimate of the amount of collagen. Other study found that inhaled ABH also reversed bronchial hyper responsiveness in a model of live guinea pigs with allergic sensitization to ovalbumin [29].

A possible limitation is that exacerbated patients received at least one dose of systemic corticosteroids prior to the extraction of the blood sample, which could influence arginase activity. However, a study in human polymorphonuclear cells found that arginase I activity is not affected by dexamethasone in vitro [11]. Another limitation is one regarding the effects of smoking on inflammation. There is a marked regulation in the expression of arginase I in murine models of exposure to tobacco, depending on time and dose [15]. Likewise, an increase in the expression of arginase I and ornithine decarboxylase (ODC) was found in the epithelium and in the bronchial smooth muscle in smoking asthmatic patients [30]. It was also determined that nicotine significantly increases the expression of mRNA of arginase I epithelial cell cultures. However, we have not established differences within each group in terms of smoking status.

With the trend of arginase activity value, a greater number of patients would be significantly different in stable COPD, compared to healthy subjects. In this matter, previous studies regarding COPD patients and arginine metabolism yielded significant results even with a scarce number of patients [31, 32].

## Conclusions

Arginase activity is elevated during an exacerbation of COPD, and this rise could be related to an increase in the production of IL-6. Levels of IL-6, IL-8, and arginase remain elevated in patients with COPD during 3 months after hospital care for exacerbation, indicating a possible persistence of underlying inflammation.

## Data Availability

The datasets analyzed during the current study are available from the corresponding author on reasonable request.

## Abbreviations

COPD: Chronic obstructive pulmonary disease
CRP: C-reactive protein
IL: Interleukin
Th1: Helper T cells type 1
Th2: Helper T cells type 2
IFN-γ: Interferon-gamma
TNF-∝: Tumor necrosis factor-∝
NOS: Nitric oxide synthase
iNOS: Inducible NOS
CD3ζ: CD3zeta chain of the T lymphocyte receptor
mMRC: Modified dyspnea scale of the Medical Research Council
CAT: COPD Assessment Test
ADMA: Asymmetric dimethyl arginine
SDMA: Symmetric dimethyl arginine
ABH: 2 (S) -amino-6-boronohexanoic acid
LPS: Lipopolysaccharide
ODC: Ornithine decarboxylase

## References

[1] Barnes P J. Mechanisms in COPD differences from asthma. Chest 2000;117(2_suppl):10S-14S. 10.1378/chest.117.2_suppl.10S. 10.1378/chest.117.2_suppl.10s 10673467

[2] Del Puerto-Nevado L, Pérez-Ria P, Girón-Martínez A, Peces-Barba G. Papel de la inflamación en la etiopatogenia de la EPOC. Arch Bronconeumol 2010; 46(Supl.11):2–7. 10.1016/S0300-2896(10)70055-7 21316554

[3] Piehl-Aulin K, Jones I, Lindvall B, Magnuson A, Abdel-Halim SM (2009). Increased serum inflammatory markers in the absence of clinical and skeletal muscle inflammation in patients with chronic obstructive pulmonary disease. Respiration.

[4] Barnes PJ, Shapiro SD, Pauwels RA (2003). Chronic obstructive pulmonary disease: molecular and cellular mechanisms. Eur Respir J.

[5] Wang Z, Zheng T, Zhu Z, Homer R, Riese R, Chapman H (2000). Interferon gamma induction of pulmonary emphysema in the adult murine lung. J Exp Med.

[6] Martínez García MA, Soler Cataluña JJ. EPOC y bronquiectasias. Arch Bronconeumol 2010; 46(Supl.3): 11–7. 10.1016/S0300-2896(10)70021-1 20620687

[7] Wedzicha JA, Seemungal TA (2007). COPD exacerbations: defining their cause and prevention. Lancet.

[8] Hurst JR, Donaldson GC, Perera WR, Wilkinson TM, Bilello JA, Hagan GW (2006). Use of plasma biomarkers at exacerbation of chronic obstructive pulmonary disease. Am J RespirCrit Care Med.

[9] Karadag F, Karul AB, Cildag O, Yilmaz M, Ozcan H (2008). Biomarkers of systemic inflammation in stable and exacerbation phases of COPD. Lung.

[10] Luiking YC, Ten Have GA, Wolfe RR, Deutz NE (2012). Arginine de novo and nitric oxide production in disease states. Am J Physiol Endocrinol Metab.

[11] Munder M, Mollinedo F, Calafat J, Canchado J, Gil-Lamaignere C, Fuentes JM (2005). Arginase I is constitutively expressed in human granulocytes and participates in fungicidal activity. Blood.

[12] Munder M. Arginase: an emerging key player in the mammalian immune system. Br J Pharmacol;158(3):638–51. 10.1111/j.1476-5381.2009.00291.x PMC2765586 19764983

[13] Ochoa JB, Bernard AC, O`Brin WE. Arginase I expression and activity in human mononuclear cells after injury. Ann Surg 2001; 233: 393–97. 10.1097/00000658-200103000-00014 PMC1421256 11224628

[14] Tadié JM, Henno P, Leroy I, Danel C, Naline E, Faisy C (2008). Role of nitric oxide synthase/arginase balance in bronchial reactivity in patients with chronic obstructive pulmonary disease. Am J Physiol Lung Cell Mol Physiol.

[15] Maarsingh H, Pera T, Meurs H (2008). Arginase and pulmonary diseases. Naunyn-Schmiedberg’s Arch Pharmacol.

[16] Global Strategy for Diagnosis, Management, and Prevention of COPD—2019. Disponibleen. https://goldcopd.org/gold-reports/.

[17] Bestall JC, Paul EA, Garrod R, Garnham R, Jones PW, Wedzicha JA (1999). Usefulness of the Medical Research Council (MRC) dyspnoea scale as a measure of disability in patients with chronic obstructive pulmonary disease. Thorax.

[18] P.W. Jones, G. Harding, P. Berry, I. Wiklund, W-H. Chen and N. Kline Leidy. Development and first validation of the COPD Assessment Test. Eur Respir J 2009, 34: 648–654. 10.1183/09031936.00102509 19720809

[19] Miller MR, Hankinson J, Brusasco V, Burgos F, Casaburi R, Coates A (2005). Series “ATS/ERS task force: standardisation of lung function testing”: Standardisation of spirometry. Eur Respir J.

[20] Garcia Navas, R. Papel inmunosupresor y citotóxico de la arginasa I y la disponibilidad de L-arginina en el sistema inmune y cáncer. Tesis doctoral. Salamanca. Centro de Investigación del Cáncer – CSIC, Facultad de Medicina, Universidad de Salamanca; 2014.

[21] Darcy CJ, Minigo G, Piera KA (2014). Neutrophils with myeloid derived suppressor function deplete arginine and constrain T cell function in septic shock patients. Crit Care.

[22] Gutierrez P, Closa D, Piñer R, Bulbuena O, Menéndez R, torres A. Macrophage activation in exacerbated COPD with and without community-acquired pneumonia. Eur Respir J 2010; 36: 285–291. 10.1183/09031936.00118909 20032016

[23] Ruzsics I, Nagy L, Keki S, Sarosi V, Illes B, Illes Z (2016). L-arginine pathway in COPD patients with acute exacerbation: a new potential biomarker. COPD.

[24] Molnar T, Pusch G, Nagy L, Keki S, Berki T, Illes Z (2016). Correlation of the L-arginine pathway with thrombo-inflammation may contribute to the outcome of acute ischemic stroke. J Stroke Cerebrovasc Dis.

[25] Scrimini S, Pons J, Agustí A, Soriano JB, Cosio BG, Torrecilla JA (2013). Differential effects of smoking and COPD upon circulatin myeloid derived suppressor cells. Respir Med.

[26] Diehl S, Rincón M (2002). The two faces of IL-6 on Th1/Th2 differentiation. Mol Immunol.

[27] Kishimoto T (1989). The biology of interleukin-6. Blood.

[28] Pera T, Zuidhof AB, Smit M, Menzen MH, Klein T, Flik G (2014). Arginase inhibition prevents inflammation and remodeling in a guinea pig model of chronic obstructive pulmonary disease. J Pharmacol Exp Ther.

[29] Maarsingh H, Zuidhof A (2008). Arginase inhibition protects against allergen-induced airway obstruction, hyperresponsiveness and inflammation. Am J Respir Crit Care Med.

[30] Bergeron C, Boulet LP, Page N, Laviolette M, Zimmermann N, Rothenberg ME (2007). Influence of cigarette smoke on the arginine pathway in asthmatic airways: increased expression of arginase I. J Allergy Clin Immunol.

[31] Jonker R, Deutz NE, Erbland ML, Anderson PJ, Engelen MP (2016). Alterations in whole-body arginine metabolism in chronic obstructive pulmonary disease. Am J Clin Nutr.

[32] Zinellu A, Fois AG, Sotgia S, Sotgiu E, Zinellu E, Bifulco F et al. Arginines plasma concentration and oxidative stress in mild to moderate COPD. PLoS ONE 2016;11(8):e0160237. 10.1371/journal.pone.0160237 PMC4968788 27479314

